# Gait Speed Modulations Are Proportional to Grades of Virtual Visual Slopes—A Virtual Reality Study

**DOI:** 10.3389/fneur.2021.615242

**Published:** 2021-08-25

**Authors:** Amit Benady, Sean Zadik, Gabriel Zeilig, Sharon Gilaie-Dotan, Meir Plotnik

**Affiliations:** ^1^Center of Advanced Technologies in Rehabilitation, Sheba Medical Center, Ramat Gan, Israel; ^2^School of Optometry and Vision Science, Bar Ilan University, Ramat Gan, Israel; ^3^The Gonda Multidisciplinary Brain Research Center, Bar Ilan University, Ramat Gan, Israel; ^4^Department of Neurological Rehabilitation, Sheba Medical Center, Ramat Gan, Israel; ^5^Department of Physical and Rehabilitation Medicine, Sackler Faculty of Medicine, Tel Aviv University, Tel Aviv, Israel; ^6^School of Health Professions, Ono Academic College, Kiryat Ono, Israel; ^7^UCL Institute of Cognitive Neuroscience, London, United Kingdom; ^8^Department of Physiology and Pharmacology, Sackler Faculty of Medicine, Tel Aviv University, Tel Aviv, Israel; ^9^The Sagol School of Neuroscience, Tel Aviv University, Tel Aviv, Israel

**Keywords:** virtual reality, gait speed, visual-physical conflict processing, rod and frame, subjective visual vertical, uphill and downhill locomotion

## Abstract

Gait is a complex mechanism relying on integration of several sensory inputs such as vestibular, proprioceptive, and visual cues to maintain stability while walking. Often humans adapt their gait to changes in surface inclinations, and this is typically achieved by modulating walking speed according to the inclination in order to counteract the gravitational forces, either uphill (exertion effect) or downhill (braking effect). The contribution of vision to these speed modulations is not fully understood. Here we assessed gait speed effects by parametrically manipulating the discrepancy between virtual visual inclination and the actual surface inclination (aka visual incongruence). Fifteen healthy participants walked in a large-scale virtual reality (VR) system on a self-paced treadmill synchronized with projected visual scenes. During walking they were randomly exposed to varying degrees of physical-visual incongruence inclinations (e.g., treadmill leveled & visual scene uphill) in a wide range of inclinations (−15° to +15°). We observed an approximately linear relation between the relative change in gait speed and the anticipated gravitational forces associated with the virtual inclinations. Mean relative gait speed increase of ~7%, ~11%, and ~17% were measured for virtual inclinations of +5°, +10°, and +15°, respectively (anticipated decelerating forces were proportional to sin[5°], sin[10°], sin[15°]). The same pattern was seen for downhill virtual inclinations with relative gait speed modulations of ~-10%, ~-16%, and ~-24% for inclinations of −5°, −10°, and −15°, respectively (in anticipation of accelerating forces). Furthermore, we observed that the magnitude of speed modulation following virtual inclination at ±10° was associated with subjective visual verticality misperception. In conclusion, visual cues modulate gait speed when surface inclinations change proportional to the anticipated effect of the gravitational force associated the inclinations. Our results emphasize the contribution of vision to locomotion in a dynamic environment and may enhance personalized rehabilitation strategies for gait speed modulations in neurological patients with gait impairments.

## Introduction

Walking is a complex process that requires specific adaptations when transitioning to inclined surfaces (1–4). These adaptations are thought to be regulated by an *Internal Model of Gravity* (IMG) that is composed of three input components (5–8): *proprioception, vestibular* (together known as body-based), and *visual* inputs. Body-based inputs are sensitive to real gravitational forces exerted on the body when walking on inclined surfaces. Visual inputs however, are assumed to be influenced by top-down expectations likely based on prior visual experience during walking on inclined surfaces (4, 9). The sensory reweighting mechanism suggests that each of the sensory inputs has a specific “weight.” The weighted inputs are added up to produce a behavioral modulation. Reweighting of the cues is constantly taking place with respect to the relevancy of each modality afferent cues (6, 10). In real life, visual and body-based inputs are typically synchronized and provide congruent information about the overall sensory experience, except for in very rare situations [e.g., the train illusion (11)]. Therefore, to evaluate the contribution of visual inputs to locomotion there is a need to artificially manipulate them in an independent manner from the body-based inputs and examine their relative “weight.” While reweighting changes have been broadly described under steady-state conditions, evidence is lacking regarding the dynamics of reweighting following transitions to various intensities of sensory inputs during locomotion. To that end, we used a novel paradigm recently presented by our lab, which allows dissociating visual inputs from body-based inputs (4, 9). We transitioned the visual scene's apparent inclination independently of the physical inclination of the treadmill. This was done using a fully immersive VR system where participants walked on a treadmill that was operated in a self-paced mode and were presented with virtual visual scenery projected on a large dome shaped screen. Our previous studies show that while walking on a leveled treadmill (i.e., 0° inclination), uphill virtually visually simulated transition of 10° is followed by a temporary increase in gait speed, while downhill virtually visually simulated transition of 10° is followed by a temporary decrease in gait speed. These visually guided gait speed modulations represent the *exertion* and *braking* effects seen in physical uphill and downhill walking, respectively. During uphill walking, the exertion effect counteracts the gravitational forces that would eventually bring a freely moving body to a stop, allowing an individual to maintain a roughly stable walking speed, typically slower than their self-selected speed during leveled walking (3, 12, 13). For downhill walking, the braking effect prevents uncontrolled speeding up (as would occur in the case of a freely moving body) and allows the individual to descend in a stable walking speed, either faster or slower than the self-selected speed on a leveled surface (3, 12, 14).

Visual field dependence is deemed as the level of reliance on visual inputs in comparison to body-based inputs (15, 16). The rod and frame test is a common method to assess visual field dependency, which is considered to evaluate the extent of subjective misperception of visual verticality (17–19). Visual field dependency varies across healthy individuals (20, 21), and it has been suggested to be higher in patients and populations with balance related disorders (22–25).

Previous studies examined gait speed modulations to inclinations of ±10°. Yet, it is unknown whether the extent of speed modulation is proportionally related to the degree of virtual visual scene transition. In the present study we aim to demonstrate that the predictions of gravitational force related consequences while walking on a slope mediated merely by visual cues are subtle and take into account the steepness of the slope. In other words, that the internal model of gravity quantifies the visual information with reference to its physical dimensions. To that end we parametrically manipulated the virtual visual scene between −15° to +15° in steps of 5° (i.e., ±15°, ±10°, ±5° and 0°), while the treadmill either remained leveled or transitioned uphill/ downhill. Our main hypothesis was that gait speed modulation is proportional to virtual visual inclination slopes during incongruent walking conditions (i.e., provoking visually induced braking (during downhill) or exertion (during uphill) effects). Specifically, given the expected gravity induced acceleration forces acting upon the body when walking on actual inclined surfaces, we anticipated smaller visually induced braking/exertion effects for smaller slopes (±5°) as compared to the effect expected when walking under the illusion of bigger slopes (±15°). We defined an additional objective for this study, to confirm our previous observation (9), that the magnitude of visual modulation on gait speed during virtual surface inclination changes of 10 degrees varies across people and is related to the individual's subjective visual misperception of verticality, as measured by the rod and frame test.

## Materials and Methods

### Participants

Fifteen young healthy adults (mean age ± SD: 27.45 ± 4.1 years old, 8 men) participated in this study. Exclusion criteria (confirmed through a questionnaire prior to recruitment) were physical restrictions, visual problems, sensorimotor impairments, or cognitive and psychiatric conditions that could potentially affect locomotion or the capability to comply to instructions. The Institutional Review Board for Ethics in Human Studies at the Sheba Medical Center, Israel, approved the experimental protocol (Approval Number 9407–12) and all participants signed a written informed consent prior to entering the study. Three participants are lacking data in the treadmill leveled vision −15° condition, and one participant is lacking data in the treadmill leveled vision −5° condition. There were four values missing from the analyses, two due to technical problems and two were excluded as outliers (more than three standard deviations from the average).

### Apparatus

The different experimental apparatuses were elaborately described in our previous work (4, 9). Herein is a brief description:

#### Virtual Reality System

Experiments were conducted in a fully immersive virtual reality (VR) system (CAREN High End, Motek Medical, The Netherlands) containing a moveable platform with six degrees of freedom. A treadmill that operates in self-paced mode, allowing participants to adjust treadmill speed to preferred gait speed, was embedded in the moveable platform (26).

#### VR Version of the Rod and Frame Test

We used the rod and frame paradigm published by Bagust et al. (20) in a VR format, which was based on the original computer screen rod and frame test (27). This test is commonly used to estimate subjective visual verticality misperception. Specifically, the test measured how visual perception of the orientation of a central bar (rod) is influenced by the orientation of a peripheral visual reference frame around it. It was implemented in our lab using Unity software and C# scripting. The participants sat upright wearing VR glasses (HTC VIVE, New Taipei City, Taiwan) and were told not to move or tilt their heads during the test. The VR environment consisted of a white frame (~16°*16°) at a certain orientation and a white rod (~11° long) inside it with its own orientation, both presented on a black background (refresh rate of 90 Hz). A sequence of 28 trials was presented during which the frame was initially rotated (relative to a vertical line) to one of seven possible random orientations: 0/±10/±20/±30 degrees (0 was vertical, + was clockwise), and each of these initial frame positions was presented four times. The initial rotation angle of the rod was also random (sampled from 0–180 degrees range distribution). The participants' task was to rotate the rod until it becomes perpendicular to the true horizon (i.e., vertical) while ignoring the surrounding frame. This was achieved using the VR system's remote control that allowed the participants to rotate the rod around its center in a clockwise or counterclockwise direction while the surrounding frame was unchanged. When the participants perceived the rod's orientation as vertical, they were supposed to respond by pressing a button on the remote control, after which the display was cleared and another trial immediately began.

### Procedure

#### VR Rod and Frame Test

The first part of the experiment after filling the informed consent was the rod and frame test. After assuring that the participant felt comfortable with the VR head mount device (HMD), a short practice trial was conducted to confirm that the participant understood the task and then the 28 test trials began. The test was not limited in time and typically lasted 10 min, including the practice trial.

#### Gait Trials in a Large-Scale VR System

##### Habituation Period to Walking in Self-paced Mode During Leveled and Inclined Surfaces

**Figure 1:**
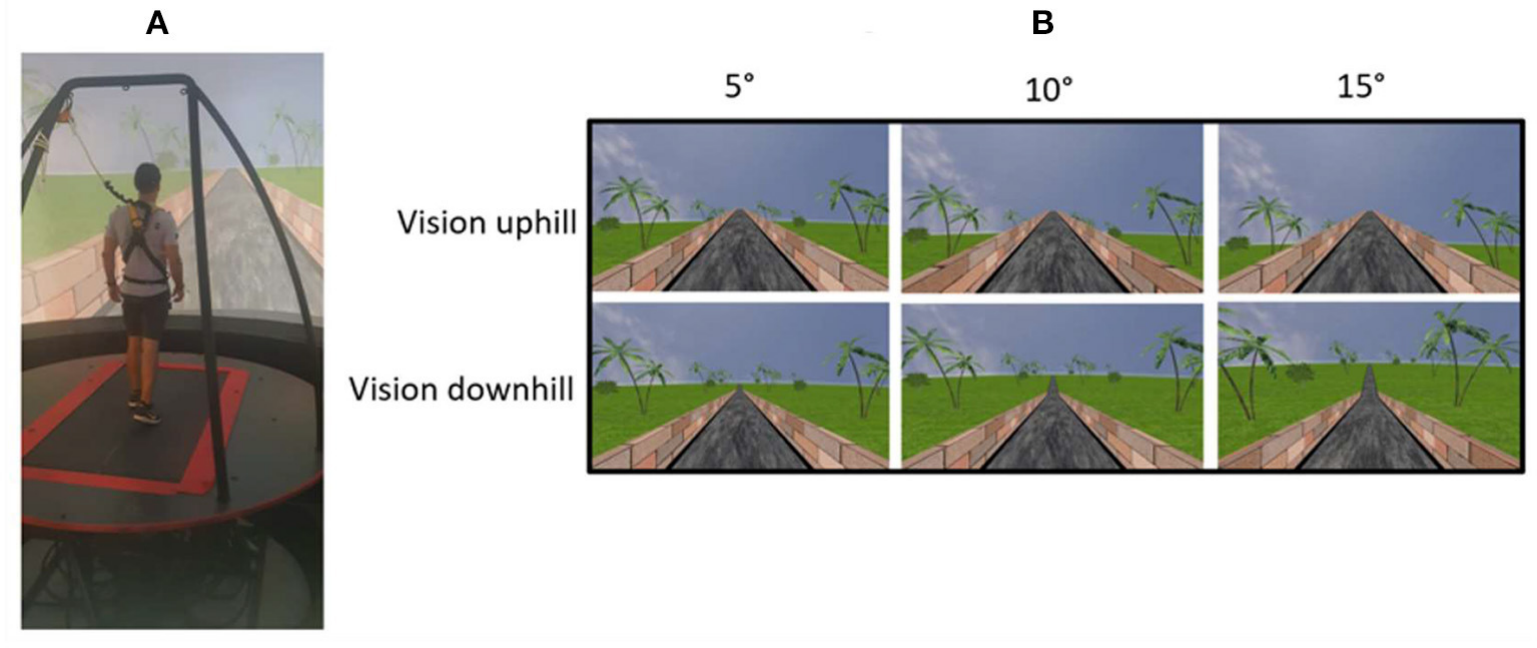
Apparatus **(A)** and visual scene manipulations **(B)**. **(A)** A participant in the VR system. A fully immersive virtual reality system containing an embedded treadmill synchronized with projected visual scenes, wherein this example the treadmill is leveled and the vision is leveled (T_L_V_V_). **(B)** Main visual manipulations regardless of the treadmill inclination. Following leveled walking and after reaching steady state velocity (SSV) and maintaining it for 12 s, a transition (5 s) occurred to one of fifteen different conditions presented in random order, lasting 65 s, in which the inclination of the treadmill (T) and/or visual scenes (V) transitioned to +5°, +10°, or +15° uphill (_U_), remained leveled (_L_), or transitioned to −5°, −10°, or −15° downhill (_D_). Visual scene inclination effect was achieved by the road appearing above (uphill), below (downhill) or converging (leveled) with the line of the horizon and in addition, the peripheral greenery is exposed more (downhill) or less (uphill) by the road. Upper row represents visual scene uphill and lower row visual scene downhill. First, second and third columns represent transition of ±5°, ±10°, and ±15°, respectively.

The participant was secured by a safety harness to a metal frame on the moveable platform (Figure 1). The first part of the habituation included familiarizing the participant with the self-paced mode of the treadmill which involved 10–15 min of leveled walking, with practicing to decrease and to increase speed until he/she mastered the walking. In the second part of the habituation, the participant performed three walking trials, one of each of the three possible inclinations (i.e., leveled, uphill and downhill walking, at this order) when the visual and the gravitational cues were synchronized (“congruent” conditions; see more details below). Each trial lasted 3–4 min.

##### Gait Experiments

The participants were informed that they would perform several gait trials, each lasting several minutes, with around 20 second intervals between them. They were instructed to walk “as naturally as possible” and were told that inclination changes may occur during walking. All trials in all conditions began from a standstill position and participants began walking with both the treadmill and the visual scene leveled until reaching steady state velocity, after which a 5 s long transition of the treadmill and/or visual scene occurred. Post transition, participants walked for additional 65 s until the treadmill slowed down and stopped. By convention, we refer to the transition start time as time zero (*t* = 0).

##### Experimental Conditions

The protocol included fifteen experimental conditions which the participant encountered in a random order and comprised of combinations of the treadmill and visual scene inclinations. Each condition started with leveled walking until the participant reached steady state velocity (SSV) and maintained it for 12 s. Following that, a transition of 5 s occurred to one of the fifteen different conditions, lasting 65 s. The treadmill (T) either transitioned uphill (U) to +5° (T_U5_)/+10° (T_U10_), remained leveled (L) at 0°, or transitioned downhill (D) to −5° (T_D5_)/−10° (T_D10_), and due to safety purposes the treadmill was not transitioned to ±15°. The visual scene (V) transitioned to +5°, +10°, or +15° uphill (V_U5_, V_U10_, V_U15_), remained leveled at 0° (L) or transitioned to −5°, −10°, or −15° downhill (V_D5_, V_D10_, V_D15_). Treadmill and visual scene *congruent* conditions that served as baseline were (i) leveled (T_L_*V*_L_), (ii) uphill (T_U5_*V*_U5_), or (iii) downhill (T_D5_*V*_D5_) walking. Note that in terms of civil engineering guidelines a 10° walking slope is considered a steep inclination for walking (28). Treadmill-visual scene *incongruent* conditions included the following visual scene manipulations: for the leveled treadmill, vision was +5° (T_L_V_U5_), +10° (T_L_V_U10_), +15° (T_L_V_U15_), −5° (T_L_V_D5_), −10° (T_L_V_D10_) or −15° (T_L_V_D15_). “*Double*” *incongruent* walking conditions included: For treadmill uphill at 5°, vision was downhill at −5° (T_U5_V_D5_), for treadmill uphill at 10°, vision was downhill at −10° (T_U10_V_D10_) or −15° (T_U10_V_D15_). For treadmill downhill at −5°, vision was uphill at +5° (T_D5_V_U5_), for treadmill downhill at −10°, vision was uphill at +10° (T_D10_V_U10_) or +15° (T_D10_V_U15_). Figure 1 depicts the experimental setup and the virtual visual scene experimental manipulations.

### Predictions of Gait Speed Modulations

A free body placed on an inclined surface is influenced by two gravitational force components, one perpendicular to the surface which is balanced by the normal reaction force, and another parallel force acting on the body in parallel to the surface and pushing the body downhill (assuming no friction or other external forces). The parallel force acting on the body is given by Fp = g ^*^ sin(α), where g is the gravitational acceleration and α is the slope angle. Since the sin function is relatively linear for small degrees (including in the range of [−15°, +15°]), such that sin(A^*^α) ≈ A^*^sin(α) (provided that |A^*^ α | <15°), the anticipated parallel forces are relatively linear, and therefore we hypothesized that the magnitude of the visually triggered braking and exertion effects will be proportional to the slopes (i.e., steeper slope—stronger anticipated forces—higher exertion/braking effect). Specifically, if X stands for the exertion effect measured in a virtual 10° slope, we predicted exertion effects of 0.5X for virtual inclinations of 5° and 1.5X for virtual inclinations of 15°. Similar predictions were made for negative virtual slopes with braking effects.

### Outcome Measures

The outcome measures were elaborately described in our previous work (4, 9). Below is a brief description:

#### Gait Speed Related Variables

To assess the post transition effects on gait speed we assessed (i) the magnitude of the peak/trough of gait speed relative to the steady state velocity (SSV; presented in %); and (ii) the time of this peak from the start of transition (seconds). Gait speed was estimated directly from a tachometer in the treadmill motor that provides the velocity signals from the treadmill belts. For full derivation of these parameters see Supplementary Methods.

#### Standardized Response to Virtual Inclination

To compute this metric, we used data from the incongruent T_L_V_U10_, T_L_V_D10_, T_D10_V_U10_, T_U10_V_D10_ conditions, similar to our previous study (9). For each participant the absolute values of the maximal relative (i.e., percent) change with respect to the SSV were calculated.

*Calculation of ratio of gravity induced behavior*- We first calculated the Area Under the Curve (AUC) separately for every second between 1s to 60s from a free body's velocity V(t) and walking speed (WS) of the congruent uphill and downhill walking conditions. Then we defined the ratio: *R* = (AUC(WS_i_)/AUC(V_(t)t = i_))^*^100. The index i refers to the time (in seconds) post-transition, with zero being the start of the transition. The equation is multiplied by 100 to avoid extremely small numbers. The ratio quantifies the extent to which WS estimates the velocity of a free body. A positive ratio indicates that both parameters were in the same direction (either accelerating or decelerating), while a negative ratio indicates the opposite direction. A ratio further from zero indicates greater gravitational influence on walking.

#### Subjective Verticality Misperception Index

For each trial the degree of deviation of the rod from the true vertical was measured and recorded as the rotation error. For each participant the mean rotation error for each of the 7 frame angles was calculated (20). We defined the rod and frame individual index to be the average angle of deviation of the rod from the true vertical when the frame was projected at ±20 degrees (8 trials in total, 4 trials of +20° and 4 trials of −20°). This parameter allows for evaluation of individual differences in gravitational misperception.

### Statistical Analyses

Values are represented by their group mean values (± SE). We used the repeated measures General Linear Model (GLM) to analyze groups of related dependent variables (amplitude and timing of the gait peaks) that represent different measurements of the same attribute (i.e., gait speed modulation). We defined two within-subject factors for the GLM analyses. The first GLM factor was the *condition class* (4 levels: T_L_V_U_, T_L_V_D_, T_D_V_U_, T_U_V_D_), and the second GLM factor was the visual inclination (3 levels: 5°,10°,15°). We further conducted GLM contrasts attempting to reveal the source of any effects that were observed in the GLM analyses. For testing how our estimates predicted true behavior, two-way repeated measures ANOVA was used. This was done by comparing observed values vs. predicted values (see section on predictions for gait speed modulations above) for treadmill leveled conditions with vision up, down or leveled. For all the above, a p-value equal or lesser than 0.05 was considered significant. Pearson correlation coefficient was computed to evaluate the relationship between (i) predicted and measured values and (ii) subjective verticality misperception index and the standardized response to virtual inclination (see *outcome measures* for more details). As the subjective verticality misperception index was compared between two distinct studies (see **Figure 7**), we transformed the indices into Z-scores so the results would be comparable.

## Results

For the averaged gait speed magnitude peak, the GLM showed a significant effect for the condition class (factor 1), [*F*_(3,36)_ = 36.07, *p* < 0.001] and for the visual inclinations (factor 2), [*F*_(2,24)_ = 23.79, *p* < 0.001]. The model also revealed a significant condition class^*^visual inclination interaction [*F*_(6,72)_ = 10.79, *p* < 0.001]. The following contrasts were found to be statistically significant: (1) For the condition class factor, T_L_V_U_ vs. T_U_V_D_ [*F*_(1,12)_ = 64.98, *p* < 0.001]; (2) For the visual inclination factor, 5° conditions were significantly different from both 10° conditions [*F*_(1,12)_ = 27.53, *p* < 0.001] and from 15° conditions [*F*_(1,12)_ = 45.08, *p* < 0.001]; (3) For the condition class^*^visual inclinations interaction, T_L_V_U_ and T_U_V_D_ showed significant interactions between the 5° and 10° visual inclinations [*F*_(1,12)_ = 36.33, *p* < 0.001] and between 5° and 15° visual inclinations [*F*_(1,12)_ = 19.13, *p* = 0.001]. For the averaged gait speed peak time, the GLM showed no significant difference between the condition class levels (factor 1), [*F*_(3,10)_ = 2.59, *p* = 0.110] and between the visual inclinations (factor 2), [*F*_(1,12)_ = 2.23, *p* = 0.150].

### Gait Speed Modulations by Visually Induced Inclinations at Leveled Treadmill

**Figure 2:**
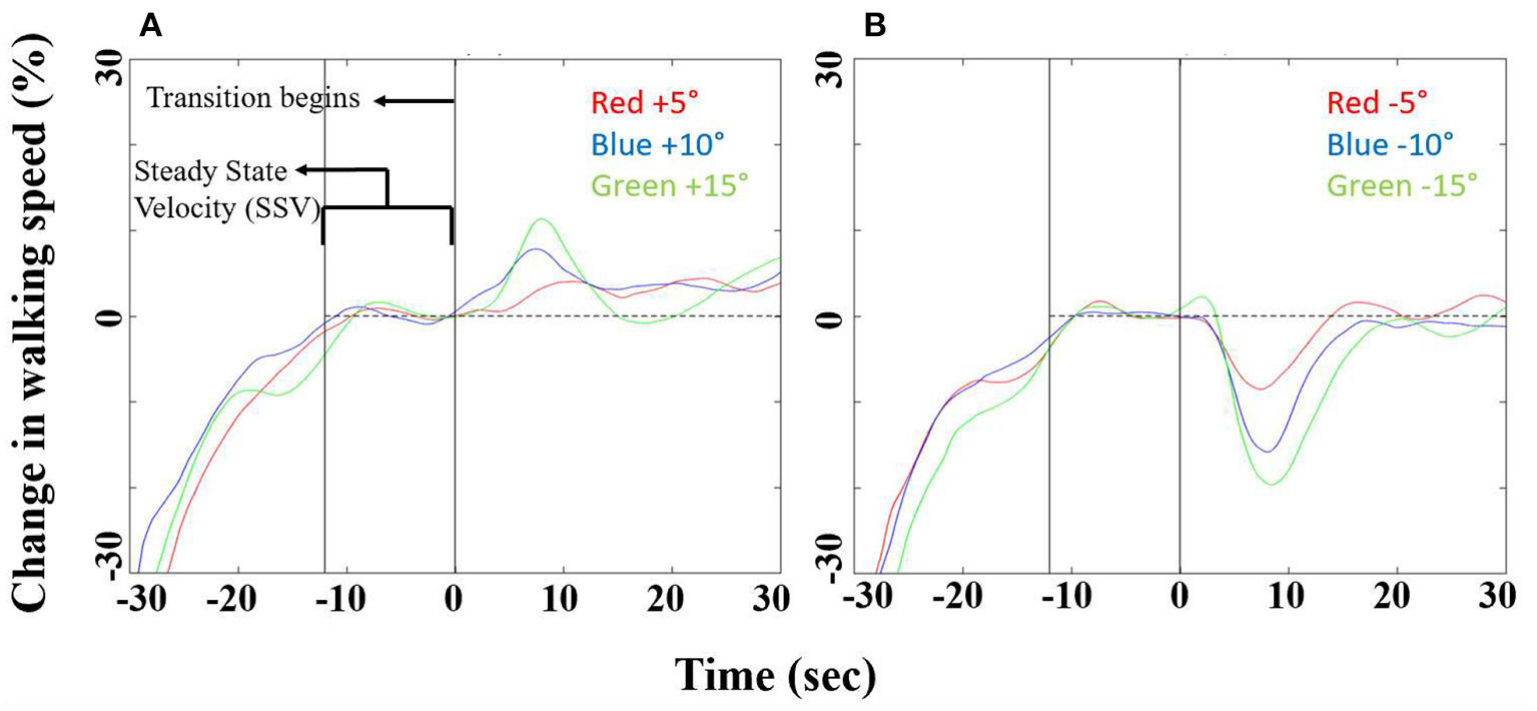
Walking speed modulations following uphill (virtually induced exertion effect) and downhill (virtually induced braking effect) virtual inclinations (*N* = 15). Speed modulations before and after **(A)** uphill or **(B)** downhill virtual inclination changes (leveled treadmill, in red for ±5°, in blue for ±10°, and in green for ±15°). X-axis represents time (seconds), 30 s pre- and post-transition. Y-axis represents the relative change in gait speed from steady state. See text for details on statistical comparisons.

In order to address our main hypothesis that gait speed modulations are linearly correlated to the degree of visual inclination, we measured gait speed while treadmill was leveled and visual scene inclination was set to +5°, +10°, +15° (Figure 2A), −5°, −10°, or −15° (Figure 2B). Figure 2 depicts the averaged relative change in gait speed from steady state velocity for each of these conditions in a time window of 60 s centered around the transition. The mean magnitude peaks ±SE of walking speed changes following virtually induced uphill slopes (the exertion effect, Figure 2A) were 6.7 ± 0.9% for +5°, 10.9 ± 1.5% for +10°, and 17.2 ± 1.7% for +15°. The mean values (±SE) of peak relative changes of walking speed changes in response to virtually induced downhill slopes (the braking effect) were −9.4 ± 2.3% for −5°, −15.8 ± 2.6% for −10° and −23.7 ± 2.7% for −15°. The timing of the gait speed peaks after transition were not significantly affected by virtual positive inclinations (mean ± SE): V_U5_ = 9.3s ± 4.5s, V_U10_ = 8.2s ± 3.7s, V_U15_ = 8.9s ± 1.7s), or by virtual negative ones (mean ± SE):V_D5_ = 8.2s ± 3.8s, V_D10_ = 8.3s ± 1.0s, V_D15_ = 8.4s ± 1.1s.

### Predicted vs. Measured Virtually Induced Exertion and Braking Effects

**Figure 3:**
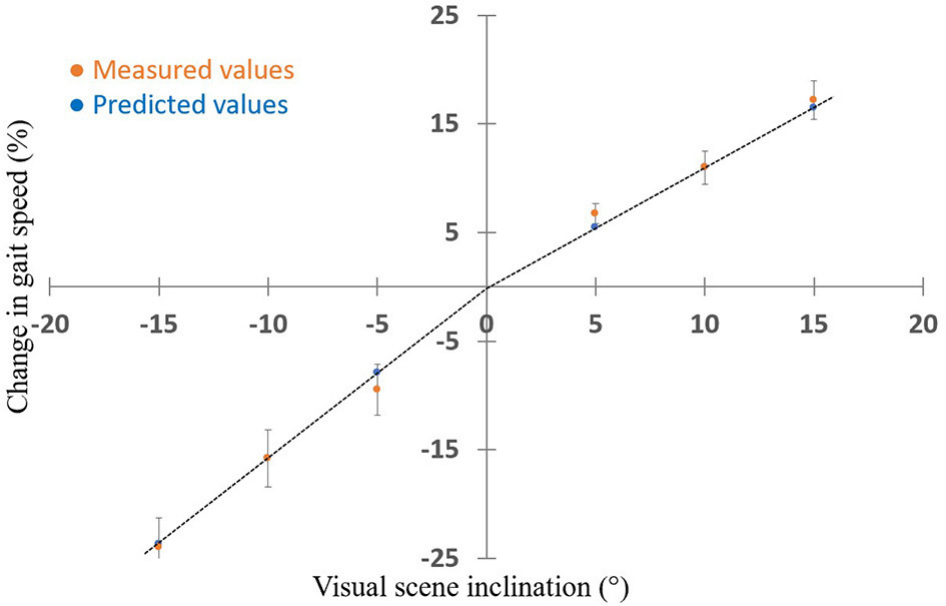
Predicted measures closely match measured gait speed at various virtual inclinations (*N* = 15). X-axis represents the virtual inclination of the visual scene (°); in all conditions the treadmill remained leveled. Y-axis represents the relative change in gait speed from the steady state (%). Orange and blue circles represent measured and predicted values (see Methods for calculation of predicted values), respectively. Error bars represent the standard error for the measured values. Dashed lines represent the slope for the predicted values. Two-way ANOVA showed no significant difference between predictive and measured values. These results support our hypothesis that the gait speed modulation is linear in this range of inclinations. Note that the positive and negative predictions are both linear with different slopes.

**Figure 4:**
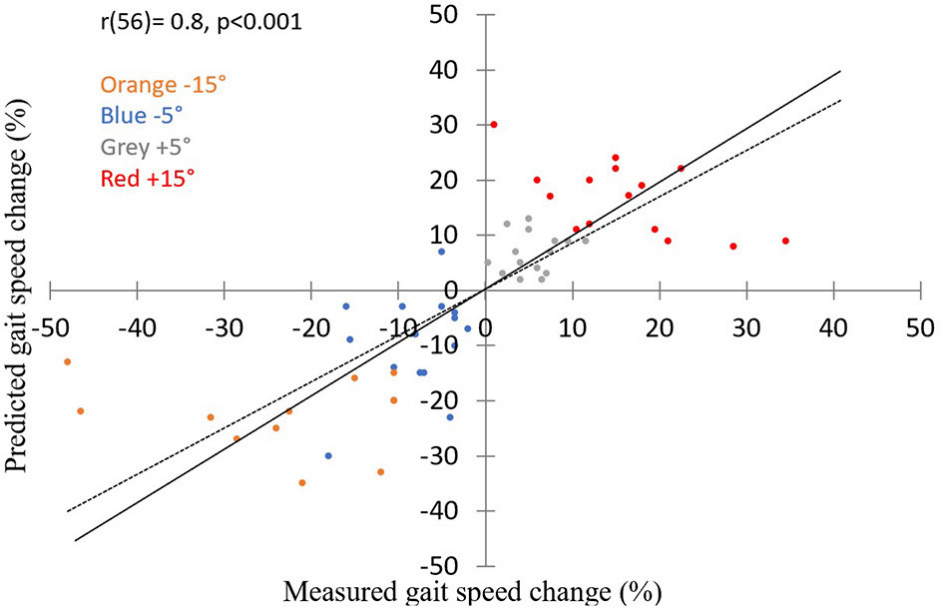
Predicted and measured changes in walking speed correlate during virtual inclinations at leveled treadmill (*N* = 15). X-axis represents the measured gait speed change from the steady state (%); Y-axis represents the predicted gait speed change from the steady state (%). For each color, a circle represents one participant (orange, blue, gray, and red circles depict T_L_V_D15_, T_L_V_D5_, T_L_V_U5_, and T_L_V_U15_, respectively). Thus, each participant is represented by 4 circles. Predicted gait speed is highly correlated with measured gait speed across virtual scene inclinations. Pearson correlation (*r* = 0.8, *p* < 0.01). Solid line represents the unity line, dashed line represents the regression line. Note that the data points are scattered roughly equal below and above the unity line.

To test our hypothesis that the extent of walking speed modulation by virtually inclined surfaces is dependent on the slope size, we estimated the expected speed modulation for slopes of ±5° and ±15° based on the results of ±10° slopes, and compared these estimates to the real modulations. Figure 3 shows how the expected results closely matched the measured ones. A two-way repeated measures ANOVA with inclination (−5, −15, 5, 15) and predicted vs. measured as factors revealed, as expected, a significant effect of inclination [*F*_(3,33)_ = 0.968, *p* < 0.001] and substantiated our observation that the predicted measures were not significantly different from the measured ones [*F*_(1,11)_ = 0.001, *p* = 0.914]. No interaction was found [*F*_(3,33)_ = 0.146, *p* = 0.684]. Furthermore, a high correlation [*r*_(13)_ = 0.8, *p* < 0.01] was seen in an individual analysis between the expected and the measured results across the four virtual inclinations measured (Figure 4).

### Relative Change in Gait Speed for Double Incongruent Walking Conditions

**Figure 5:**
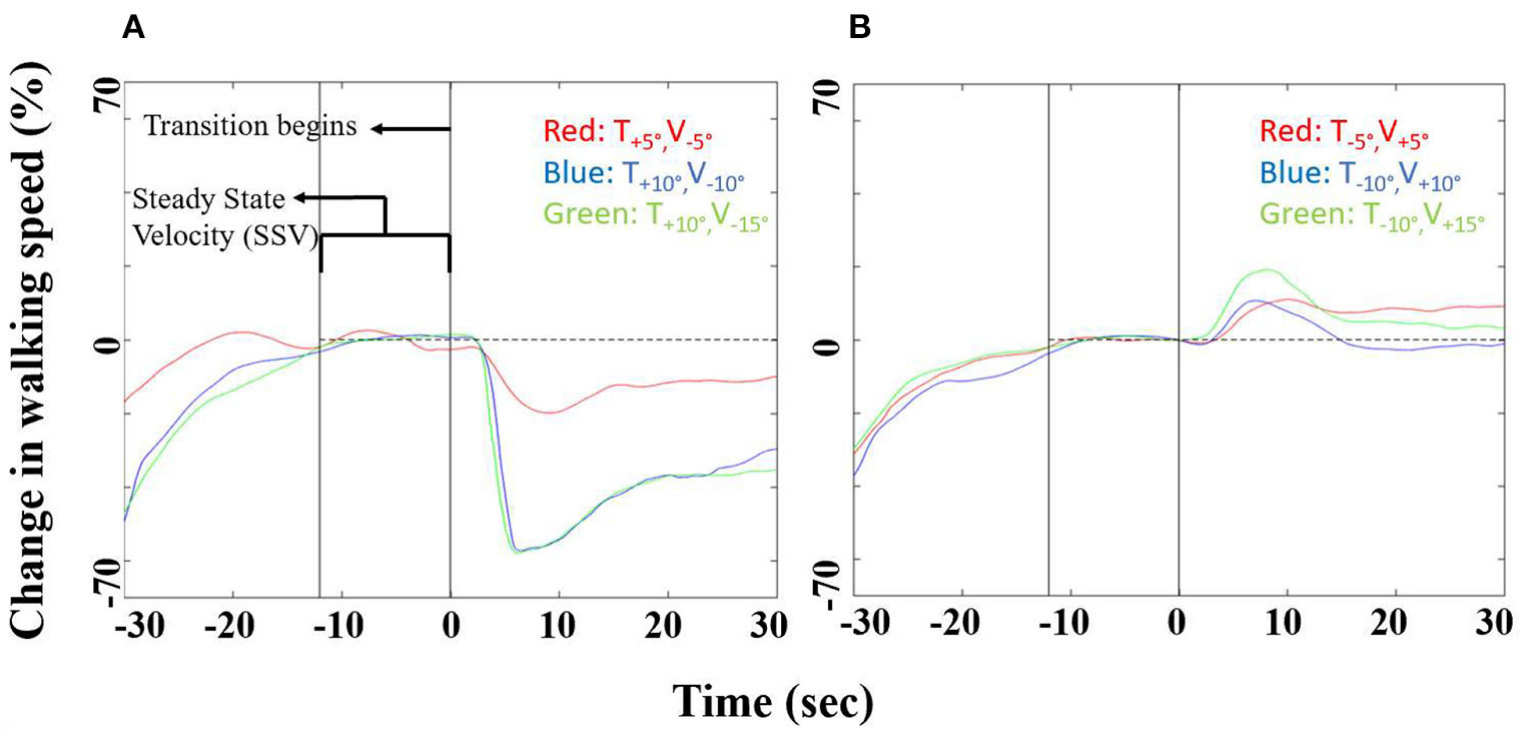
Gait speed modulations in the double in congruency walking conditions (*N* = 15). Speed modulations before and after **(A)** treadmill up vision down or **(B)** treadmill down vision up walking conditions (red lines for ±5°, blue lines for ±10°, and in green lines for treadmill ±10° and vision ±15°). X-axis represents time (seconds), 30 s pre- and post- transition. Y-axis represents the relative change in gait speed from steady state (%). See text for details on statistical comparisons.

In addition to measuring virtually simulated exertion and braking effects, we also manipulated both the treadmill and the visual scene in different directions to further examine the role of vision during walking. Figure 5A presents the walking speeds during conditions where the treadmill transitioned upward with inclinations of +5° or +10°, while the visual scene transitioned downward with inclinations of −5°, −10°, or −15°, respectively. Figure 5B presents the same concept but in the opposite direction, where the treadmill transitioned downwards, and the visual scene transitioned upwards. The mean magnitude peaks ±SE of walking speed changes following the transition (treadmill up, Figure 5A) were −23 ± 3% for T_U5_V_D5_, −60 ± 7% for T_U10_V_D10_ and −63 ± 7% for T_U10_V_D15_. The mean magnitude peaks ±SE of walking speed changes following the transition (treadmill down, Figure 5B) were 13 ± 3% for T_D5_V_U5_, 9 ± 4% for T_D10_V_U10_ and 20 ± 3% for T_D10_V_U15_. The timing of gait speed peaks after transition to uphill treadmill inclinations while the vision transitioned downward were (mean ±SE): T_U5_V_D5_ = 8.47s ± 2.97s, T_U10_V_D10_ = 7.87s ± 3.14s, T_U10_V_D15_ = 9.23s ± 4.55s and for downhill treadmill inclinations while the vision transitioned uphill were (mean ± SE): T_D5_V_U5_ = 11.7s ± 4.9s, T_D10_V_U10_ = 9.42s ± 4.0s, T_D10_V_U15_ = 9.51s ± 3.99s.

### Ratio of Gravity-Induced Behavior in Congruent Uphill and Downhill Walking Conditions

**Figure 6:**
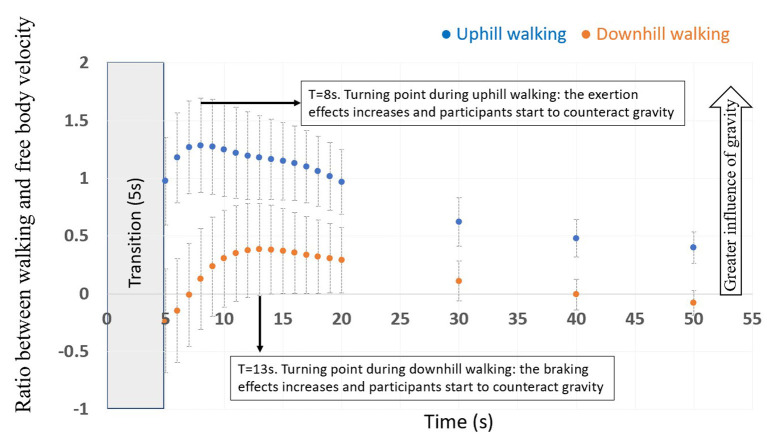
Ratio of gravity-induced behavior in uphill and downhill walking. The average ratio between gait speed and velocity of a free-moving body over time for ±5° downhill and uphill congruent conditions (*N* = 15), error bars represent standard error. The turning points represent the time (seconds) in which the participants applied the exertion effect to counteract gravitational deceleration in uphill walking (8 s) and conversely, applied the braking effect to counteract gravitational acceleration in downhill walking (13 s).

Our next step was to compare the changes in exertion and braking effects over time (post-transition). To that end, we computed the normalized ratio between the areas under the curve of gait speed for the ±5° congruent conditions [for ±10° see Cano Porras et al. (4)], divided by free body velocity at the same inclination [i.e., V(t) = g^*^sin±5^*^t] (Figure 6). The higher the ratio (i.e., further from zero), the stronger the effect of gravity and the weaker the exertion and braking effects are. The analysis revealed a robust differential response to gravity in uphill vs. downhill walking (*p* = 0.034), reflecting a weaker accelerating influence of natural gravity and a strong braking effect. Yet, both uphill and downhill walking initially showed an increasing ratio, which suggests increasing natural gravity influence. For uphill walking, the turning point occurred at 8 s, while for downhill walking the turning point occurred at 13 s from the transition. Beyond this point participants expended more effort (the exertion effect) or braked themselves (the braking effect) to maintain gait speed during uphill and downhill walking, respectively.

### Relation Between Visual Modulation of Gait Speed During Visual-Physical Incongruent Conditions and Subjective Misperception of Verticality

**Figure 7:**
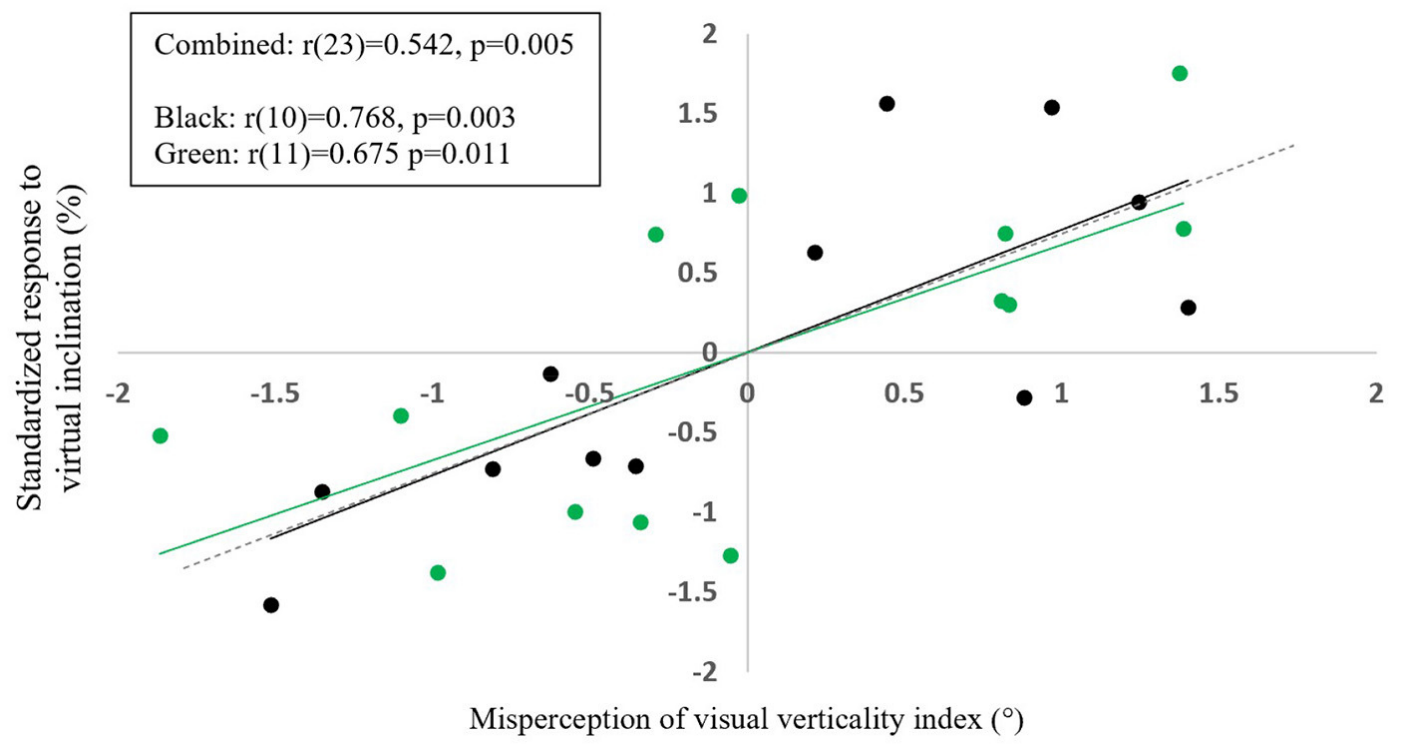
The extent of visual dependence during locomotion on inclined surfaces is linked to misperception of visual verticality. X axis represents the z-score for misperception of visual verticality index as assessed by the rod and frame test; Y axis represents the z-score for standardized response to ±10° virtual inclination based on incongruent walking conditions. Each circle represents one participant obtained from two separate experiments with the same protocol [green (*N* = 13) and black (*N* = 12)]. A significant correlation was found between these measures either for both cohorts combined: *N* = 25, *r* = 0.542, *t*_(23)_ = 3.09, *p* = 0.005, and for each cohort separately (i) *N* = 13, *r*_(11)_ = 0.675, *t*_(11)_ = 3.03, *p* = 0.011, (ii) *N* = 12, *r*_(10)_ = 0.768, *t*_(10)_ = 3.79 *p* = 0.003. The dashed line represents the combined linear regression line (Y = 0.71X + 6E−16), the black and green line represent the regression lines of each cohort separately as can be seen the 3 regression lines almost overlap.

Finally, we hypothesized that the magnitude of visual modulation on gait speed varies across people and may be related to the individual's visual field dependency. Therefore, for each participant we calculated the magnitude of visual modulation on gait speed and the visual field dependence index. The former was calculated based on the changes in gait speed for the ±10° incongruent conditions (see Methods) and the latter based on the rod and frame test which estimates the visual field dependence. As can be seen in Figure 7, which combines data from two separate studies with the same protocol (9), when we compared these 2 measurements together we found that they were significantly correlated (*r* = 0.542, *p* = 0.005), and this was also the case when each subgroup was measured separately. This indicates that people with higher visual field dependency are likely to have stronger walking speed modulations during virtual inclination changes, suggesting that these processes may rely upon associated mechanisms. No correlation was found between the changes in gait speed for inclinations of ±5° and ±15° and the index of subjective visual misperception of verticality estimated by the rod and frame test.

## Discussion

### Summary of Findings

In this study we investigated how gait speed is modulated by the visual virtual inclination slopes. As hypothesized, we showed that when a larger conflict was created between the sensory inputs, larger visually induced braking and exertion effects were measured (Figure 2), as expressed by gait speed modulations. The response-intensity relations of the visually induced braking and exertion effects were linear and proportional to the theoretical gravity induced tangential downhill and uphill acceleration levels, respectively (Figures 3, 4). We did not find any difference in the timing of the peak/trough of gait speed modulations when different virtual inclination levels were introduced. Furthermore, as previously described (9) we observed that the inter-subject variability in the virtually induced braking and exertion effects (at ±10°) can be explained in part by the individual's visual field dependency as measured by the rod and frame test (20).

### The Relative Weight of Visual Cues During Locomotion Modulations

Although we found a significant linear effect for visual inputs on gait speed modulations while walking at physical leveled inclinations when only the visual scene was transitioned, we did not find an effect on the timing of the gait speed peaks across the virtual visual induced transitions, suggesting that the timing of the peak effect of visually induced braking and exertion effects is similar regardless of the inclination. This result points us to two mechanisms regarding human locomotion: (i) indirect prediction, which is a process controlling locomotion patterns based on accumulated experience, promptly activating pre-programmed gait patterns in the presence of a perturbation (in our case the transition to an inclined slope). (ii) Sensory reweighting which is an iterative mechanism of recalibration of the relevant cues. This finding demonstrates that the sensory reweighting processes (6, 10, 29) which balance between vision (which in this case is influenced by a “faked” visual incline), and body-based cues (which are influenced by the physical treadmill inclination) occur at similar times, independent of the intensity of the sensory input conflict (for the discrepancy levels used in the present study). Adapting the hypothesis that the central nervous system (CNS) is using a weighted summation model of the sensory cues in execution of motor control, we assume that when there is a conflict between the inputs, the more reliable cues become more heavily weighted. In other words, the visual cues are likely to affect the indirect prediction mechanism (29) in proportion to the conflict size created by the visual inclination, but the time it takes for the sensory reweighing to “kick in” is apparently constant for virtually induced uphill walking (~8.8 s) and for virtually induced downhill walking (~ 8.2 s). This study strengthens the sensory reweighting theory by showing that once this mechanism governs, quantitative estimates of sensory weights are modulated regardless of the amplitude of perturbations provided by visual manipulations. To further compare the weight of vision contribution in uphill and downhill walking, we conducted two types of double incongruent setups (T_U_V_D_, T_D_V_U_). In both T_U_V_D_ and T_D_V_U_ conditions, the vision did not significantly affect gait speed. In conclusion, visual inputs predominantly affect gait speed during leveled (neutral) inclinations proportionally to the degree of virtual visual inclination. The proportional weight of visual inputs is reduced when gravitational cues (physical inclination) oppose the virtual visual cues.

### Inter Participant Variability

The relation between subjective visual vertical (SVV) that is assumed to represent visual field dependency (17–19) and postural stability in the healthy population is well established (15, 30–32). Yet, the locomotive responses, which are behaviorally expressed by changes in gait speed is not fully understood. While the present finding confirms earlier findings with this specific paradigm (at ±10°) (9), it is unclear why these findings were not generalized to the other inclinations (±5° or ±15°). These seemingly conflicting results might be explained by assuming that visual field dependency varies across healthy individuals within a certain range (9, 20, 21). In extreme scenarios where the conflict is up to 15° (e.g., treadmill 0° and vision +15°), it may be that visual field dependence no longer plays a role in affecting perception, which may explain why no correlation was observed between the rod and frame test and locomotive response. Although we did find a behavioral change in virtually induced inclinations of ±15°, which was relatively bigger than inclinations of ±10°, we could not predict those changes by the rod and frame index. These behavioral changes may suggest that in cases with extreme discrepancy, the integration of sensory cues follows another mechanism. A possible mechanism could be directed to instinctively prevent us from falling backwards while walking uphill (or in the case of the visual scene transitioning downward, decreasing the speed to maintain stability and prevent forward acceleration). The same was true when the conflict was small (i.e., virtually induced inclination of ±5°). We suggest that humans adapt their gait speed according to the virtually induced inclination, but the mechanism relies on different sources rather than the traditional visual dependence. This analog was also seen in our analysis of the rod and frame index itself, as well as for Bagust et al. (9, 20). When the frame was tilted at ±30°, or when the frame was not tilted (0°), no significant change was seen across individuals. The only difference was seen when the frame was tilted at ±10° and ±20°. These findings suggest that the visual dependence, as measured by the rod and frame index is not susceptible to extreme discrepancies.

### Exertion and Braking Effects as Measured in Congruent Conditions

The braking and exertion effects were previously quantified in our lab for congruent inclinations of ±10° (4). Here we quantified the modulations related to inclinations of ±5° (Figure 6). Our results confirm the role of vision in the initiation of the exertion effect during real uphill walking, since the timing of the deflection point (i.e., turning point, which symbols the time that took the participants to accommodate to the new inclination) (*t* = 8 s) was almost similar to the peak of the gait speed increase in response to virtual uphill walking (9.3 s). Overall, the accommodation to uphill walking is faster for smaller inclinations (5°, *t* = 8.0 s) as compared to greater inclinations (10°, *t* = 11.0 s). These findings are consistent with the notion that people try to minimize their energetic cost while walking (29, 33), and thus when walking on steeper inclination, more energy is used and it will take more time to reach the new steady state (34). For downhill walking at −5°, the braking effect started at 13 s post transition. Surprisingly, the trough of the virtually induced braking effect was at 8.2 s. This time difference indicates that when no sensory discrepancy exists, the accommodation time to a downhill slope is longer, emphasizing the role of body-based cues to maintain locomotion and adjust the body according to the gravitational laws of physics. We also note that subtle influences of the self-pace control parameters employed in this experiment may interfere with these estimations (e.g., forward/backward translations on inclined surfaces provides reduced feedback to the speed controller as compared to leveled walking), as well as the individual ability to control gait speed alterations under this self-paced paradigm.

### Clinical Implications

As natural continuation to traditional physical therapy base interventions, VR advantages were emphasized in light of its ability to incorporate motor learning principles such as real-time multisensory feedback, task variation, objective progression, and task-oriented repetitive training (35–38). VR systems allow to simulate scenarios from real life, and yet provide therapists a unique opportunity to work in a risk-free environment, and train patients in motor rehabilitation paradigms (39–41). We posit that the VR media provides additional substrates for developing new motor learning strategies. For example, targeting salient physiological processes such as *indirect prediction* and *sensory reweighting* (6, 10, 29), which are adopted by humans during the course of development and can be recruited for treating locomotion impairments. For example, Lamontagne et al. show that using optic flow manipulations, stroke patients instantaneously increased their walking speed by 44%, for comparison the healthy control group increased their speed by 32% (42). Combining these findings with Bonan et al. (22, 23), which show that stroke patients have increased visual dependency, we can suggest that people with higher visual field dependency will have greater speed modulation following an optic flow manipulation. These findings are in line with our present study (Figure 7) in a young healthy population, showing this relation between visual field dependency and the magnitude of gait speed change following an optic manipulation (either optic flow or virtual inclination transition). Decreased sensorimotor integration is an essential characteristic of neurological patients suffering from gait impairments (43). As inclined walking is part of our daily living, better understanding the physiology in young healthy populations is a first step in harnessing the present, or similar (4, 9), paradigms for clinical use. For example, as a relatively ecological tool employed for diagnosis of sensorimotor integration impairment. Such approach would imply establishing norms (of outcomes such as those presented in the present work) based on data from healthy participants. These norms can be contrasted with data recorded from participants with pathological conditions such as persons with Parkinson's disease or persons post stroke. As done in the present contribution, such potential diagnosis can address whether the graded response pattern to virtual inclination intensity is preserved in these cohorts. Along these lines, our paradigm could also evaluate the success of conventional therapies by comparing the multisensory integration pre- and post- therapies and examine whether there was any progress and to what extent. Finally, by “recruiting” this manipulation as a clinical therapy, and repeating the manipulation continuously over longer periods, it can be used to alter the impaired coupling between perception and action, and enhance gait adaptation and sensorimotor integration. Moreover, based on the present study, a new tool which unites a short walking period in a visual conflict paradigm and the rod and frame test can potentially estimate visual dependency in locomotion. Such a tool will help to identify those who may benefit from visual conflicts paradigms, hence facilitating personalized rehabilitation program. For example Brady et al. (44), showed that highly visually dependent people successfully trained to one set of visual conflicts, but were not able to apply their adapted skills to a new discordant environment in comparison to lower visually dependent people. It is yet unclear whether the gait speed modulations we found in a young healthy population will be replicated in populations with gait impairments. To the best of our knowledge, although there are studies that measured the effect of optic flow training (45), none of these paradigms have been effectively translated to clinical practice. Both theoretical and clinical studies are needed to harness the ability of VR to introduce sensory incongruence for rehabilitation benefits.

## Limitations

We note the following limitations associated with this study: (1) Treadmill inclinations of ±15° could not be included due to safety restrictions, thus limiting the ability to reach conclusions about gait speed modulations at these walking inclinations. (2) The experimental design did not include all the possible walking conditions, and by that prevented us from computing the proper statistical tests. The limiting factor was the presumed maximal number of walking trails (i.e., 15) that can be presented without causing fatigue. We included the main conditions where the treadmill is leveled and the visual scene transitioned. (3) The third limitation is related to the fact that the visual slope transition times in the incongruent conditions were always 5 s in all conditions (i.e., ±5°, ±10°, or ±15°). This means that, e.g., the 15° transition change was 3X faster than of the 5° transition. This adds a potential confounder (i.e., visual slope transition speed). We acknowledge that this point should be addressed in future studies.

## Conclusions

Virtually induced braking and exertion effects which are expressed by gait speed modulations are linearly related to the degree of virtual inclination. Furthermore, these modulations are highly correlated to the individual visual field dependency assessed by the rod and frame test while walking at virtual inclinations of ±10°. Our findings add another stratum to the understanding of sensorimotor integration during locomotion in healthy populations and has the potential to contribute to develop VR based rehabilitation strategies in the future.

## Data Availability Statement

The datasets generated for this study are available upon reasonable request from the corresponding author.

## Ethics Statement

The studies involving human participants were reviewed and approved by The Institutional Review Board for Ethics in Human Studies at the Sheba Medical Center. The participants provided their written informed consent prior to entering the study.

## Author Contributions

MP, SG-D, and AB conceptualized the study. AB and SZ collected the data. GZ supervised recruitment and ethical aspects. AB analyzed the data and was the primary writer. All authors participated in reviewing, editing, and approving the final manuscript.

## Conflict of Interest

The authors declare that the research was conducted in the absence of any commercial or financial relationships that could be construed as a potential conflict of interest.

## Publisher's Note

All claims expressed in this article are solely those of the authors and do not necessarily represent those of their affiliated organizations, or those of the publisher, the editors and the reviewers. Any product that may be evaluated in this article, or claim that may be made by its manufacturer, is not guaranteed or endorsed by the publisher.

## References

[1] Cavagna G. The Role of Gravity in Human Walking: Pendular Energy Exchange, External Work and Optimal Speed. (2000). Available online at: https://www.ncbi.nlm.nih.gov/pmc/articles/pmc2270143/ (accessed July 4, 2020). 10.1111/j.1469-7793.2000.00657.x PMC2270143 11060138

[2] Wall-Scheffler CM Chumanov BH. EMG activity across gait and incline : the impact of muscular activity on human morphology. Am J Phys Anthropol. (2011) 143:601–11. 10.1002/ajpa.21356 20623603 PMC3011859

[3] Kimel-Naor S Gottlieb A Plotnik M. The effect of uphill and downhill walking on gait parameters: a self-paced treadmill study. J Biomech. (2017) 60:142–9. 10.1016/j.jbiomech.2017.06.030 28757238

[4] Cano Porras D Zeilig G Doniger GM Bahat Y Inzelberg R Plotnik M. Seeing gravity: gait adaptations to visual and physical inclines – a virtual reality study. Front Neurosci. (2020) 13:1308. 10.3389/fnins.2019.01308 32038123 PMC6992711

[5] Merfeld DM Zupan L Peterka RJ. Humans use internal models to estimate gravity and linear acceleration. Nature. (1999) 398:615–8. 10.1038/19303 10217143

[6] Campos JL Butler JS Bülthoff HH. Contributions of visual and proprioceptive information to travelled distance estimation during changing sensory congruencies. Exp Brain Res. (2014) 232:3277–89. 10.1007/s00221-014-4011-0 24961739

[7] Lacquaniti F Bosco G Gravano S Indovina I La Scaleia B Maffei V. Multisensory integration and internal models for sensing gravity effects in primates. BioMed Res Int. (2014) 2014:1–11. 10.1155/2014/615854 25061610 PMC4100343

[8] Balestrucci P Daprati E Lacquaniti F Maffei V. Effects of visual motion consistent or inconsistent with gravity on postural sway. Exp Brain Res. (2017) 235:1999–2010. 10.1007/s00221-017-4942-3 28326440

[9] Benady A Zadik S Ben-Gal O Cano Porras D Wenkert A Gilaie-Dotan S. Vision affects gait speed but not patterns of muscle activation during inclined walking—a virtual reality study. Front Bioeng Biotech. (2021) 9:127. 10.3389/fbioe.2021.632594 33898402 PMC8062981

[10] Assländer L Peterka RJ. Sensory reweighting dynamics following removal and addition of visual and proprioceptive cues. J Neurophysiol. (2016) 116:272–85. 10.1152/jn.01145.2015 27075544 PMC4969387

[11] Seno T Fukuda H. Stimulus meanings alter illusory self-motion (vection)-experimental examination of the train Illusion. Seeing Perceiving. (2012) 25:631–45. 10.1163/18784763-00002394 23550369

[12] Sun J Walters M Svensson N Lloyd D. The influence of surface slope on human gait characteristics: a study of urban pedestrians walking on an inclined surface. Ergonomics. (1996) 39:677–92. 10.1080/00140139608964489 8854986

[13] Sinitski EH Lemaire ED Baddour N Besemann M Dudek NL Hebert JS. Fixed and self-paced treadmill walking for able-bodied and transtibial amputees in a multi-terrain virtual environment. Gait Posture. (2015) 41:568–73. 10.1016/j.gaitpost.2014.12.016 25661003

[14] McIntosh AS Beatty KT Dwan LN Vickers DR. Gait dynamics on an inclined walkway. J Biomech. (2006) 39:2491–502. 10.1016/j.jbiomech.2005.07.025 16169000

[15] Isableu B Ohlmann T Crémieux J Amblard B. How dynamic visual field dependence-independence interacts with the visual contribution to postural control. Human Movement Sci. (1998) 17:367–91. 10.1016/S0167-9457(98)00005-09187294

[16] Willey CR Jackson RE. Visual field dependence as a navigational strategy. Attention Perception Psychophys. (2014) 76:1036–44. 10.3758/s13414-014-0639-x 24519434 PMC4429127

[17] Lopez C Lacour M Magnan J Borel L. Visual field dependenceg-independence before and after unilateral vestibular loss. NeuroReport. (2006) 17:797–803. 10.1097/01.wnr.0000221843.58373.c8 16708017

[18] Isableu B Gueguen M Fourré B Giraudet G Amorim MA. Assessment of visual field dependence: comparison between the mechanical 3D rod-and-frame test developed by Oltman in 1968 with a 2D computer-based version. J Vestibular Res. (2008) 18:239–47. 19542598

[19] Bagust J. Rod and frame alignment times increase when the frame is tilted. Psychol Behav Sci. (2013) 2:66. 10.11648/j.pbs.20130202.17

[20] Bagust J. Assessment of verticality perception by a rod-and-frame test: preliminary observations on the use of a computer monitor and video eye glasses. Arch Phys Med Rehabil. (2005) 86:1062–64. 10.1016/j.apmr.2004.05.022 15895360

[21] Kaleff CR Aschidamini C Baron J de Leone CD Canavarro S Vargas CD. Semi-automatic measurement of visual verticality perception in humans reveals a new category of visual field dependency. Brazil J Med Biol Res. (2011) 44:754–61. 10.1590/S0100-879X2011007500090 21779636

[22] Bonan I Guettard E Leman MC Colle FM Yelnik AP. Subjective visual vertical perception relates to balance in acute stroke. Arch Phys Med Rehabil. (2006) 87:642–6. 10.1016/j.apmr.2006.01.019 16635626

[23] Bonan I Hubeaux K Gellez-Leman MC Guichard JP Vicaut E. Influence of subjective visual vertical misperception on balance recovery after stroke. J Neurol Neurosurg Psychiatr. (2007) 78:49–55. 10.1136/jnnp.2006.087791 17012343 PMC2117806

[24] Crevits L Venhovens J Vanoutrive J Debruyne J. False perception of visual verticality in multiple sclerosis. Eur J Neurol. (2007) 14:228–32. 10.1111/j.1468-1331.2006.01636.x 17250735

[25] Schindlbeck KA Naumann W Maier A Ehlen F Marzinzik F Klostermann F. Disturbance of verticality perception and postural dysfunction in Parkinson's disease. Acta Neurol Scand. (2018) 137:212–7. 10.1111/ane.12859 29063605

[26] Plotnik M Azrad T Bondi M Bahat Y Gimmon Y Zeilig G. Self-selected gait speed - over ground versus self-paced treadmill walking, a solution for a paradox. J NeuroEng Rehabil. (2015) 12:20. 10.1186/s12984-015-0002-z 25881130 PMC4374285

[27] Witkin HA Asch SE. Studies in space orientation IV. Further experiments on perception of the upright with displaced visual fields. J Exp Psychol. (1948) 38:762–82. 10.1037/h0053671 18893191

[28] Proffitt DR Bhalla M Gossweiler R Midgett J. Perceiving geographical slant. Psychonomic Bull Rev. (1995) 2:409–28. 10.3758/BF03210980 24203782

[29] O'Connor SM Donelan JM. Fast visual prediction and slow optimization of preferred walking speed. J Neurophysiol. (2012) 107:2549–559. 10.1152/jn.00866.2011 22298829

[30] Lord SR Webster IW. Visual field dependence in elderly fallers and non-fallers. Int J Aging Human Dev. (1990) 31:267–77. 10.2190/38MH-2EF1-E36Q-75T2 2090615

[31] BarrCMcLoughlinJvan den BergMELSturnieksDLCrottyM. Visual field dependence is associated with reduced postural sway, dizziness and falls in older people attending a falls clinic. J Nutr Health Aging. (2016) 20:671–5. 10.1007/s12603-015-0681-y27273359

[32] Lee SC. Relationship of visual dependence to age, balance, attention, and vertigo. J Phys Ther Sci. (2017) 29:1318–22. 10.1589/jpts.29.1318 28878455 PMC5574361

[33] Selinger JC O'Connor SM Wong JD Donelan JM. Humans can continuously optimize energetic cost during walking. Curr Biol. (2015) 25:2452–6. 10.1016/j.cub.2015.08.016 26365256

[34] Minetti AE Moia C Roi GS Susta D Ferretti G. Energy cost of walking and running at extreme uphill and downhill slopes. J Appl Physiol. (2002) 93:1039–46. 10.1152/japplphysiol.01177.2001 12183501

[35] Levac D Missiuna C Wishart L Dematteo C Wright V. Documenting the content of physical therapy for children with acquired brain injury: Development and validation of the motor learning strategy rating instrument. Phys Ther. (2011) 91:689–99. 10.2522/ptj.20100415 21415229

[36] Levac DE Glegg SMN Sveistrup H Colquhoun H Miller P Finestone H. Promoting therapists' use of motor learning strategies within virtual reality-based stroke rehabilitation. PLoS ONE. (2016) 11:168311. 10.1371/journal.pone.0168311 27992492 PMC5167266

[37] Levin MF Weiss PL Keshner EA. Emergence of virtual reality as a tool for upper limb rehabilitation: incorporation of motor control and motor learning principles. Phys Ther. (2015) 95:415–25. 10.2522/ptj.20130579 25212522 PMC4348716

[38] Keshner EA Fung J. The quest to apply VR technology to rehabilitation: Tribulations and treasures. In: Journal of Vestibular Research: Equilibrium and Orientation. IOS Press (2017). p. 1–5. Available online at: https://pubmed.ncbi.nlm.nih.gov/28387695/ (accessed August 30, 2020). 10.3233/VES-170610 28387695

[39] Darekar A McFadyen BJ Lamontagne A Fung J. Efficacy of virtual reality-based intervention on balance and mobility disorders post-stroke: a scoping review. J NeuroEng Rehabil. (2015) 12:1–14. 10.1186/s12984-015-0035-3 25957577 PMC4425869

[40] Cano Porras D Siemonsma P Inzelberg R Zeilig G Plotnik M. Advantages of virtual reality in the rehabilitation of balance and gait: systematic review. Neurology. (2018) 90:1017–25. 10.1212/WNL.0000000000005603 29720544

[41] de Keersmaecker E Lefeber N Geys M Jespers E Kerckhofs E Swinnen E. Virtual reality during gait training: does it improve gait function in persons with central nervous system movement disorders? A systematic review and meta-analysis. NeuroRehabilitation. (2019) 44:43–66. 10.3233/NRE-182551 30814368

[42] Lamontagne A Fung J McFadyen BJ Faubert J. Modulation of walking speed by changing optic flow in persons with stroke. J NeuroEng Rehabil. (2007) 4:1–8. 10.1186/1743-0003-4-22 17594501 PMC1913055

[43] de Dieuleveult AL Siemonsma PC van Erp JBF Brouwer AM. Effects of aging in multisensory integration: a systematic review. Front Aging Neurosci. (2017) 9:1–14. 10.3389/fnagi.2017.00080 28400727 PMC5368230

[44] Brady RA Peters BT Batson CD Ploutz-Snyder R Mulavara AP Bloomberg JJ. Gait adaptability training is affected by visual dependency. Exp Brain Res. (2012) 220:1–9. 10.1007/s00221-012-3109-5 22585123

[45] Kang HK Kim Y Chung Y Hwang S. Effects of treadmill training with optic flow on balance and gait in individuals following stroke: randomized controlled trials. Clin Rehabil. (2012) 26:246–55. 10.1177/0269215511419383 21971754

