# Supplementary material for: Gait Speed Modulations Are Proportional to Grades of Virtual Visual Slopes—A Virtual Reality Study
Source: Front Neurol. 2021 Aug 25;12:615242. doi: 10.3389/fneur.2021.615242 (PMC8425350; doi:10.3389/fneur.2021.615242)
Supplement: Supplementary file 1 [file Data_Sheet_1.docx]

**Supporting material for the *Methods* section**

The response characteristics programmed into the self-paced treadmill were fixed across all participants. The acceleration command followed the equation:

(1) X″ = PΔX−DΔX * X′

Where X" is the acceleration command to the treadmill motor, ΔX is the difference between the subjects’ position and the center of the treadmill (anterior – posterior), and X′ is the subject speed (i.e., the current speed). D and P are coefficients set by the manufacturer.

The delay in generating the acceleration command was 0.008 milliseconds (120 Hz).

As the reviewer correctly noted, since the X displacement is measured relative to a fixed frame of reference, i.e., when the X axis is parallel to the horizontal plane which is the plane of the treadmill in leveled position, the same progression/recession with reference to a fixed point on the treadmill will result in different “ΔX” when the treadmill is leveled as compared to when the treadmill is slanted, e.g., by 10 degrees. We, however, chose not to modify equation (1) during physical inclination from the following reasons: (a) since the maximal incline is 5 degrees the geometrical distortion in very small (i.e., Cosine(5deg) = 0.996); (b) the transition to different inclination lasts 5 seconds and during this time continuous modification is needed and (c) regardless of the equation, behavior dictates the speed outcome, as the participants settle in the center of the treadmill when they feel they reach the desired selected speed.

*Elaboration on Gait speed related variables*- To assess the post transition effects on gait speed we followed the methodologies introduced by Cano-Porras et al., (Cano Porras et al., 2020).

**Steady-state velocity-** A real-time algorithm monitoring treadmill speed determined steady-state velocity (SSV). According to the algorithm, SSV is attained after 1) a minimum 30s of walking, and 2) a consecutive period of 12s with gait speed coefficient of variance less than two percent. Upon satisfying both conditions, transition of treadmill and/or visual scene inclination (as appropriate for the experimental condition) was automatically triggered.

**Normalization of gait speed (Fig. S-1)-**  Normalization of gait speed (WS) in each experimental condition consisted of three steps. First, WS was divided by the averaged SSV (i.e., from the 12s that defined the SSV period). In the new trace the mean value of the 12s SSV period is 1. The ratio between WS and the SSV was presented as percentage. Finally, in order to clearly distinguish between velocity increase responses and velocity decrease responses the normalized trace was shifted so that the mean value of WS of the SSV period would be zero.


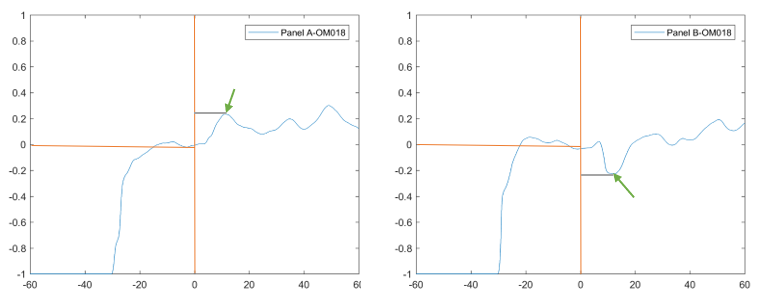


**Figure S-1.** Deriving gait speed related variables. Fig. S-1 depicts example of responses to virtual uphill (T_L_V_U_; panel A) and downhill (T_L_V_D_; panel B) transitions from one participant (OM018). Vertical orange line represents the transition, horizontal orange line represents steady state velocity. On these traces the peak/ trough were identified (green arrows) and the time from transition was calculated (grey line). Units used for magnitude were the relative change (%) in comparison to the SSV values. The second parameter is the time of maximal change (c.f., Fig. S-1). Adapted from Benady et al. (Benady et al., 2021, see main text).
